# Supplementary material for: Subcellular Feature-Based Classification of α and β Cells Using Soft X-ray Tomography
Source: Cells. 2024 May 18;13(10):869. doi: 10.3390/cells13100869 (PMC11119489; doi:10.3390/cells13100869)
Supplement: Supplementary file 1 [file cells-13-00869-s001.zip › Cells-Supplemental Information.pdf]

Supplementary Material

Cell Type	Voxel size (nm)	Cell Volume (μm³)	Nucleus Volume (μm³)	Nucleus Volume(%)	Vesicle Volume (μm³)	Vesicle Volume(%)	Vesicle Number	Vesicle Diameter (nm)	Nucleus LAC (μm⁻¹)	Vesicle LAC (μm⁻¹)	Cytosol LAC (μm⁻¹)	Mitochondria LAC (μm⁻¹)
**α_1**	43.4	627.3495663	133.9632279	21.35384084	7.036248585	1.121583398	880	233.6748328	0.21232659	0.35968328	0.23548414	0.3001793
**α_2**	30	693.004572	85.924854	12.39888703	4.310037	0.621934858	915	201.4174401	0.26021409	0.41465557	0.29144618	0.38529399
**α_3**	41.6	624.3280286	137.7806858	22.06863692	10.87831677	1.742404037	1934	248.9270089	0.19960697	0.32507855	0.24161716	0.33972824
**α_4**	41.6	357.4397363	89.62750772	25.07485839	3.608419729	1.00951835	966	192.8202457	0.24834067	0.37920013	0.28775084	0.35507408
**α_5**	41.6	224.1526752	64.55409118	28.79916162	3.326645797	1.484098191	828	189.0861683	0.25148439	0.38320458	0.27483377	0.36516342
**α_6**	39.97	475.3242422	97.53298919	20.51925413	6.644291891	1.397844103	1719	216.2323509	0.24134913	0.39139012	0.28411624	0.34116709
**α_7**	46.5	1053.416392	159.6092633	15.15158341	6.819740825	0.6473927	1610	217.9785591	0.22966661	0.36169311	0.25193512	0.35924211
**α_8**	33.1	576.075641	127.5911866	22.14833912	5.322858816	0.923986094	1842	201.2672404	0.22651798	0.38380224	0.23798567	0.4086028
**β_1**	43.4	1396.805947	107.6496822	7.706845925	7.159277074	0.512546291	2557	166.7709835	0.1939431	0.32310513	0.22953857	0.30892169
**β_2**	43.4	1493.032455	84.90257303	5.686585898	7.102708493	0.47572365	3045	154.1116635	0.2151885	0.35084963	0.23711036	0.3351267
**β_3**	33.1	742.2587326	79.30979128	10.68492532	2.228755379	0.300266643	1865	142.59702	0.26024485	0.35204035	0.27401403	0.36319551
**β_4**	41.6	914.4833318	85.61132929	9.361715661	3.962616906	0.433317565	1869	175.5660769	0.20945519	0.34599665	0.24932864	0.34501064
**β_5**	39.6	1128.038009	149.0142045	13.210034	1.904766799	0.168856615	838	156.0477278	0.20008653	0.33981138	0.21640025	0.38297018
**β_6**	45.5	1390.321163	192.4066459	13.83900721	7.380003392	0.53081285	2553	176.7484126	0.18551314	0.31683621	0.23454882	0.29869804
**β_7**	41.6	1271.432015	127.5638251	10.03308266	5.146441777	0.404775223	1963	171.0901069	0.20378827	0.30905032	0.21596016	0.31082597

Supplementary Table S1. Summary of whole α and β cell statistics.

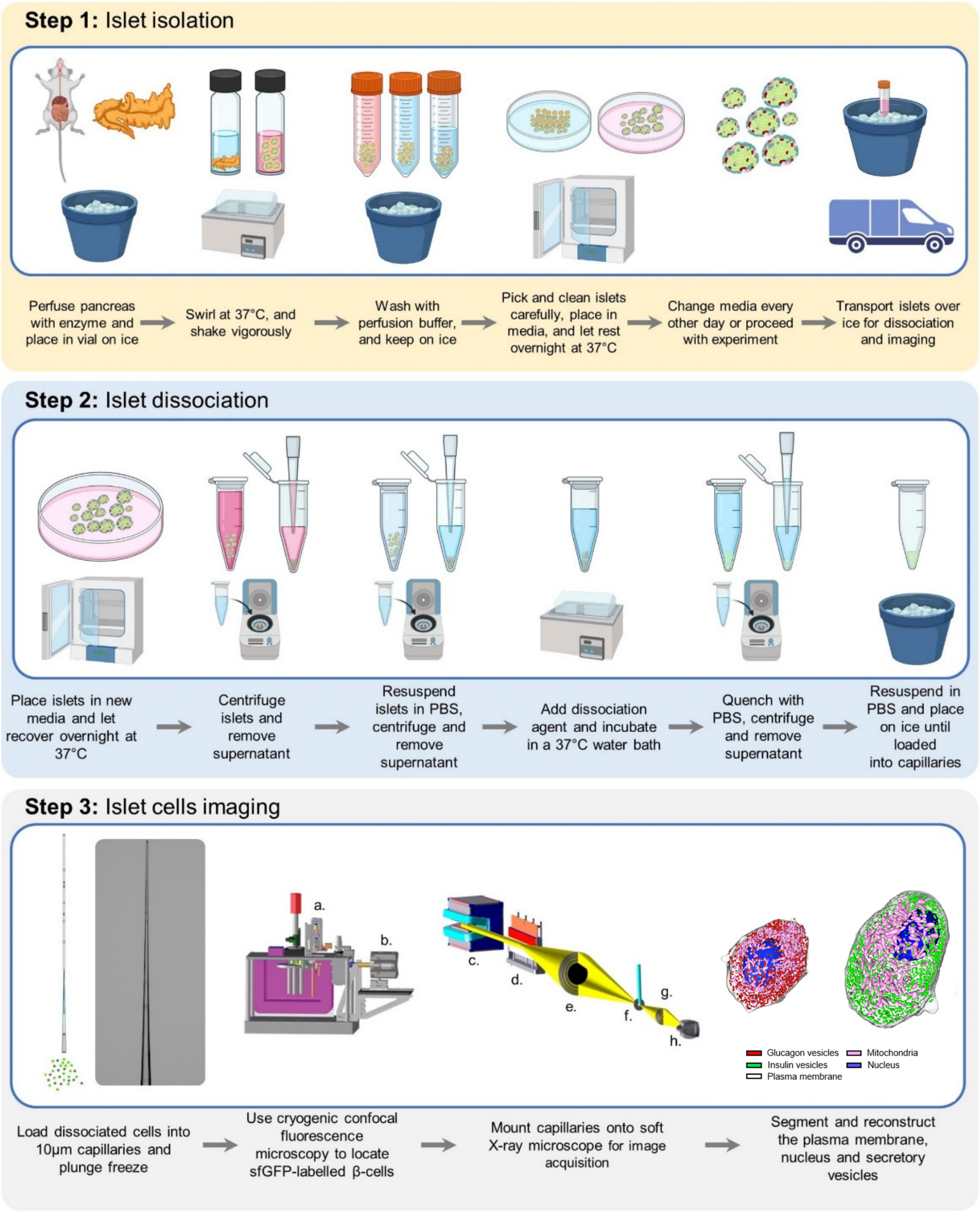

Figure S1. Pipeline for islet isolation, dissociation, and imaging. Pipeline for islet isolation, dissociation, and imaging. The top panel depicts the process of islet isolation. Islets are extracted and isolated from a transgenic mouse containing C-peptide-bearing Super folder Green Fluorescent Protein (CpepSfGFP)-labeled β cells. After islet extraction they are transported to the imaging site, the National Center for X-ray Tomography (NCXT, Lawrence Berkeley National Lab,

Berkeley, CA). The middle panel depicts the dissociation of islets into its single cells. The bottom panel depicts the loading of single cells into thin-walled glass capillaries which are rapidly plunge-frozen prior to imaging. The cell type is identified using a cryogenic confocal fluorescence microscope: (a) goniometer and sample holder; (b) detector. The 3D subcellular structure is then visualized using the soft X-ray microscope XM-2 at the NCXT. The energy range used for the imaging is selected by directing the X-ray beam coming from a bending magnet (c) onto a mirror (d); the beam is focused using a condenser zone plate (e) onto the specimen (f). A microzone plate (g) is then used to magnify the 2D projection images onto a CCD detector (h). 3D tomograms are then reconstructed, and the subcellular architecture is obtained for both cell types. (Created with BioRender.com)

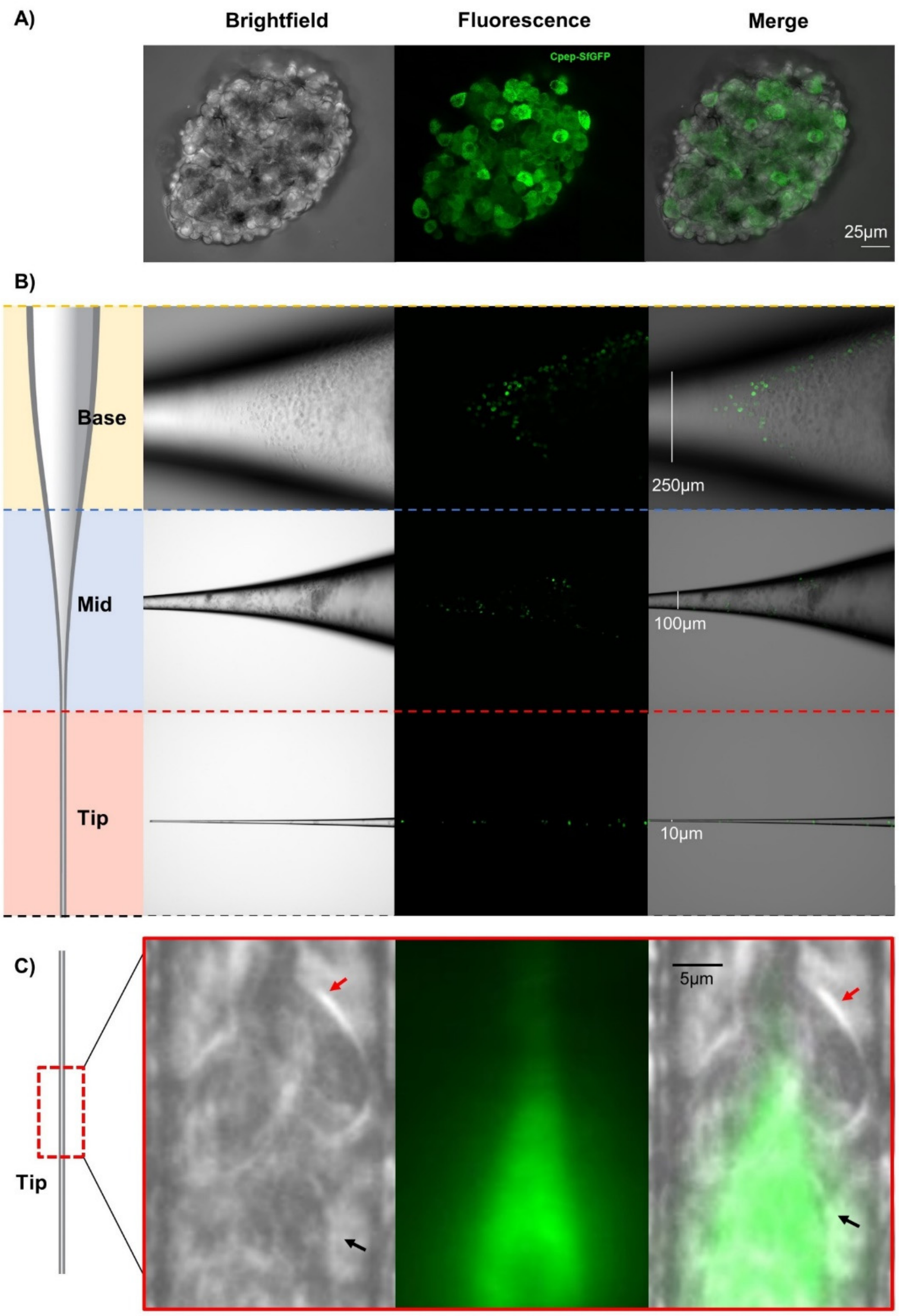

Figure S2. β Cell Identification**. A**) An islet extracted from a transgenic mouse containing CpepSfGFP-labelled β cells. Left: Brightfield image of an intact islet with no extracellular tissue attached. Middle: Fluorescence emitted by CpepSfGFP labeled β cells when excited with a 488 nm laser. The β cells are primarily localized in the center of the islet which is typical of mouse islet architecture. Right: Merged image showing the non-β cell types primarily localized in the outer ring of the islet. Scale bar: 25 μm. **B**) Confocal image of dissociated cells loaded into a microcapillary 10-μm tip. In the top panel, the base of the capillary shows a number of cells and cell clusters present along with multiple dissociated β cells visible under fluorescence (scale bar: 250 μm). In the middle panel, the diameter of the capillary reduces in size as the cells reach the mid-section, resulting in a compact packing of the cells (scale bar: 100 μm). The bottom panel shows dissociated single-cells loaded into the 10μm-13μm region of the capillary, with β cells clearly visible at the end of the tip (scale bar: 10 μm). **C**) Tracing the fluorescent signal of β cells inside the tip of the capillary. β cells (black arrows) can be distinguished from *α* cells (red arrows) in cell clusters by detecting the CpepSfGFP fluorescent signal, using a cryogenic confocal fluorescent microscope. The position of the respective cell types is marked on the capillary for further data acquisition on the soft x-ray microscope (scale bar: 5 μm).

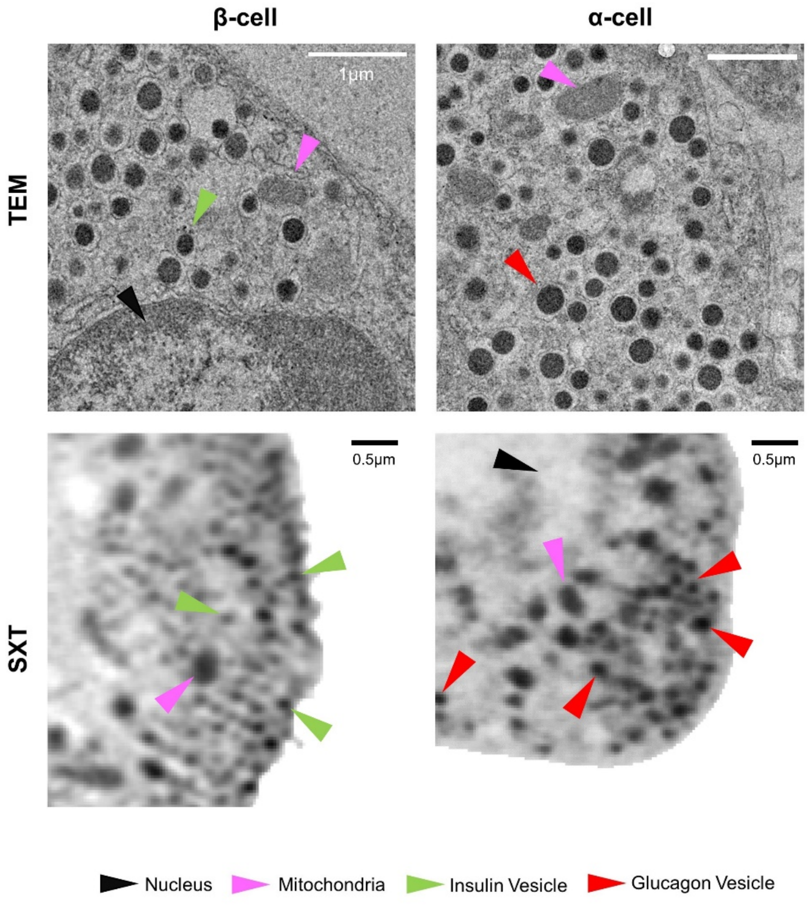

Figure S3. Comparison between vesicular morphological features in *α* and β cells in TEM and SXT data**.** Top panel: ultrathin sections of mouse islets imaged using the TEM. Organelles can be identified by their standard morphological features. Insulin vesicles, glucagon vesicles, mitochondria, and nucleus are identified by green, red, pink, and black arrowheads, respectively. Glucagon vesicles can be differentiated from insulin vesicles based on a more electron dense core along with a thinner halo around the core. Scale bar: 1 μm. Bottom panel: representative orthoslices of *α* and β cells (*α*_3 and β_6, respectively) imaged using SXT. Using this technique, vesicles cannot be distinguished between cell types on the basis of morphology and LAC values. Insulin vesicles, glucagon vesicles, mitochondria, and nucleus are

identified by green, red, pink, and black arrowheads, respectively. Based on the LAC value, insulin vesicles (0.33±0.03 μm-1) can be distinguished from glucagon vesicles (0.37±0.04 μm-1). Scale bar (TEM): 1 μm. Scale bar (SXT): 0.5 μm

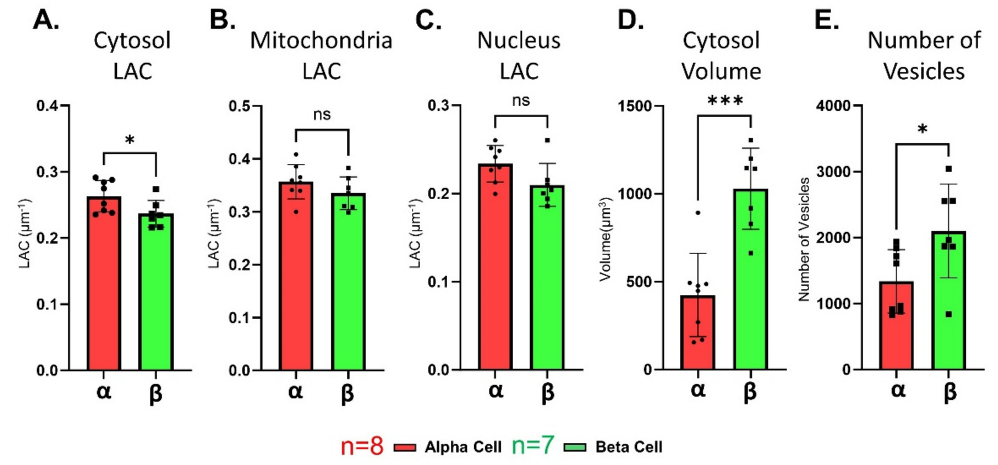

Figure S4. Quantitative analysis of α and β cell morphology. **A)** Comparison between cytosol LAC demonstrating a significantly higher value (*p=0.04) for α cells (0.26±0.02 μm^{-1}) as compared to β cells (0.24±0.02 μm^{-1}). **B-C)** Comparison between organelle LAC values showing a higher mitochondrial and nuclear LAC for α cells (mitochondria – 0.36±0.03 μm^{-1}; nucleus – 0.24±0.02 μm^{-1}) as compared to β cells (mitochondria – 0.33±0.03 μm^{-1}; nucleus – 0.21±0.02 μm^{-1}). **D)** Plot showing a significantly higher (***p=0.0002) cytosolic volume (cell volume subtracted by nuclear volume) for β cells as compared to α cells. **E)** Number of vesicles per cell type indicating a significantly higher (*p=0.04) number of secretory vesicles in β cells (2100±710) as compared to α cells (1337±480). Error bars in each plot are representative of the standard deviation. Welch's t-test was used as a statistical test. n=8 for α cells (red) and n=7 for β cells (green).

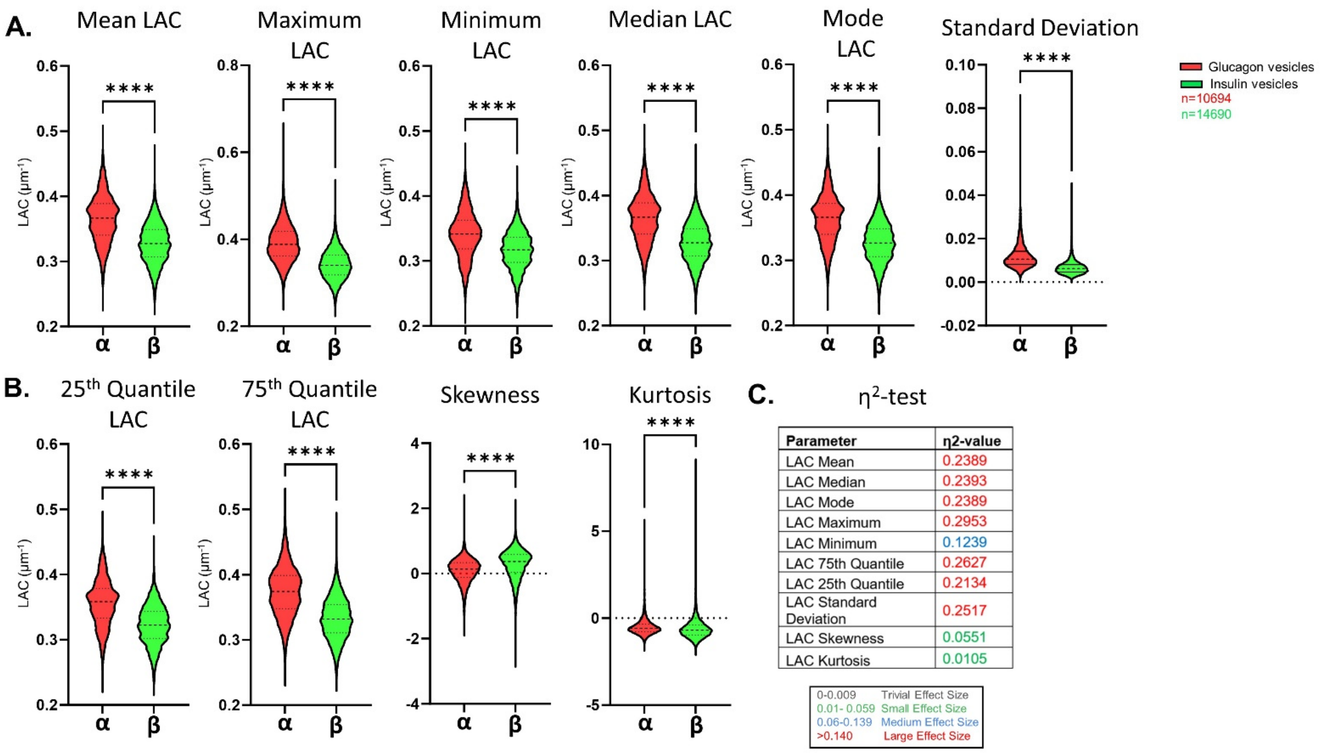

Figure S5. LAC-based differences in insulin and glucagon granules**. A)** Violin plots showing a significantly higher value of mean LAC, maximum LAC, minimum LAC, median LAC, mode LAC and standard deviation for glucagon vesicles as compared to insulin vesicles **B)** Violin plots showing significantly higher values for 25th Quantile LAC, 75th Quantile LAC and Kurtosis and significantly lower values of Skewness for glucagon vesicles as compared to insulin vesicles. **A-B)** n=10694 for glucagon vesicles (red); n=14690 for insulin vesicles (green). ****p<0.0001 for all plots; Welch's t-test. **C)** Table of effect size(η^2) values for all the LAC based parameters showing a large effect size (red) for Mean LAC, Median LAC, Mode LAC, Maximum LAC, 75th Quantile LAC, 25th Quantile LAC, and Standard Deviation. Minimum LAC has a medium effect size (blue) and Skewness and Kurtosis have small effect sizes (green). Error bars in all plots are representative of the standard deviation. n=10694 for glucagon vesicles (red) and n=14690 for insulin vesicles (green).

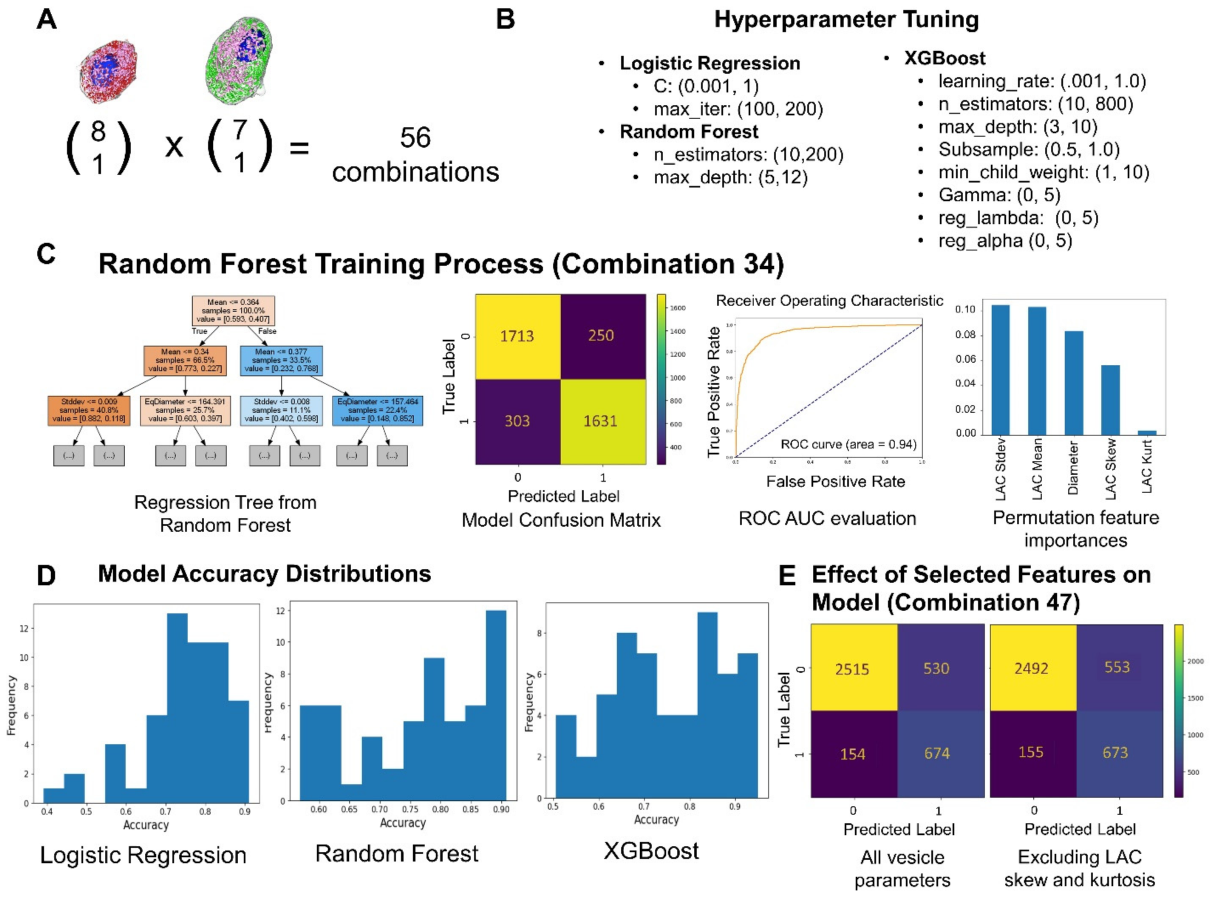

Figure S6. Machine learning training process**. A)** To calculate the number of combinations of 1 left-out α and 1 left-out β cell, the ^nC_r formula was used. **B)** Range of hyperparameters used for tuning models. Optimal parameters were evaluated based on accuracy scores and found used grid search for logistic regression/random forest and Bayesian optimization for XGBoost. **C)** Outputs at each stage of training a given model, in this case combination 34 using Random Forest. From left to right: sample regression tree for random forest, confusion matrix detailing model performance, ROC AUC Curve, and raw feature importance scores. **D)** Distribution of accuracy scores across the three models. All XGBoost and random forest scores were above 50%, but a few logistic regression scores were below this threshold. **E)** Removing LAC skewness and kurtosis features when training a XGBoost model does not significantly affect model performance.

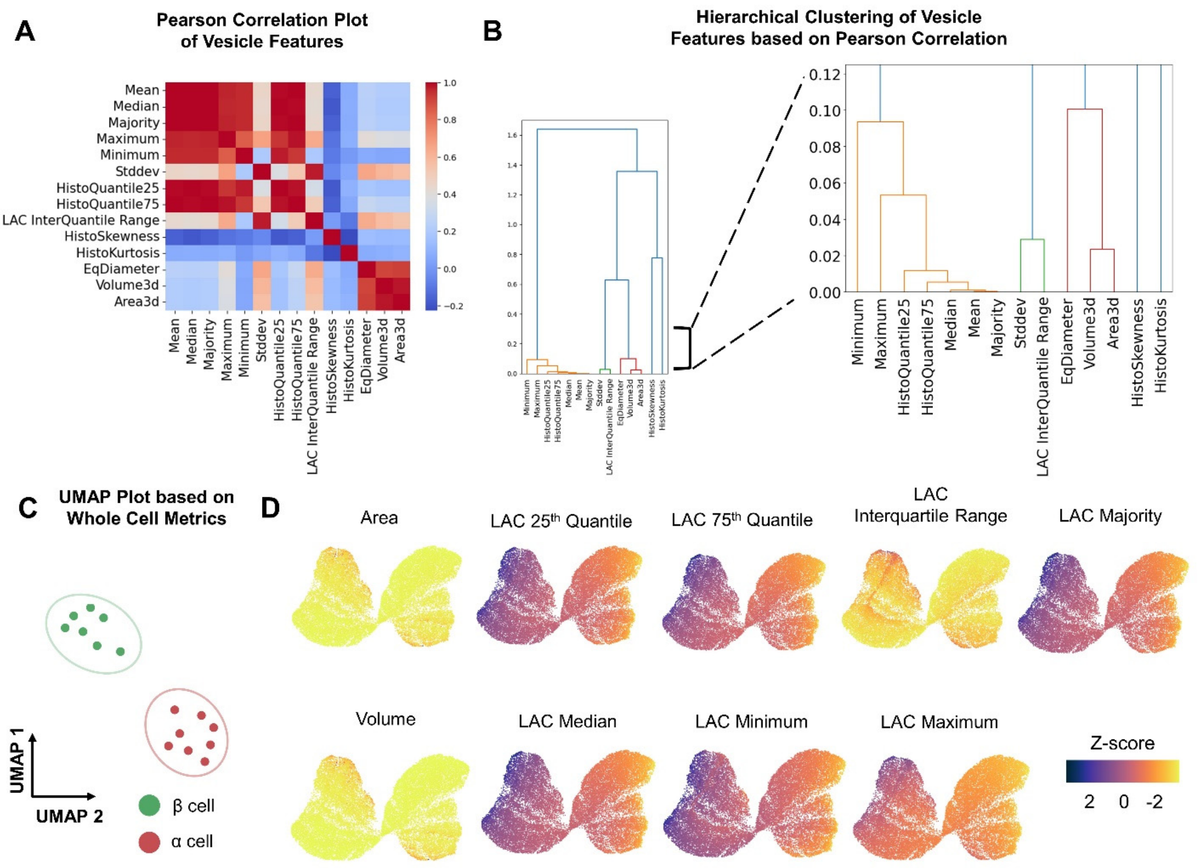

Figure S7. Addressing multicollinear feature importances. **A)** Pearson correlation plot of vesicle features. A number of distinct co-correlated regions can be observed. **B)** Hierarchical clustering based on correlation scores. Cluster cutoff threshold was decided on interpretation of the correlation plot and UMAP vesicle parameter embeddings. **C)** UMAP based on whole cell metrics from the 8 α and 7 β cells. Two structural cell clusters corresponding to each cell type can be found. **D)** UMAPs of the remaining nine vesicle metrics not selected as representative features of hierarchical clusters. Many UMAP plots represent similar trends to other highly correlated features. However, the shift in values in the LAC Maximum plot is different from the other correlated vesicle metrics. A higher proportion of vesicles have lower values relative to the average LAC maximum, which may delineate vesicle subpopulations containing very high density of biochemical cargo.
